# From concept to application: Exploring the evolution and potential of DUBTAC technology

**DOI:** 10.1016/j.apsb.2025.11.022

**Published:** 2025-11-19

**Authors:** Danfeng Wang, Wenjian Min, Binjian Jiang, Haopeng Sun, Chengliang Sun, Peng Yang

**Affiliations:** a State Key Laboratory of Natural Medicines and Jiangsu Provincial Key Laboratory of Targetome and Innovative Drugs Medicines, China Pharmaceutical University, Nanjing 211198, China; b Department of Medicinal Chemistry, School of Pharmacy, China Pharmaceutical University, Nanjing 211198, China; c Institute of Innovative Drug, China Pharmaceutical University, Nanjing 211198, China

**Keywords:** DUBTAC, Heterobifunctional molecules, Targeted protein stabilization, Ubiquitin–proteasome system, Drug discovery

## Abstract

Deubiquitinase-targeting chimeras (DUBTAC), as a highly promising emerging technology, can precisely remove ubiquitin chains from target proteins by recruiting deubiquitinases (DUBs), thereby enhancing the stability of the target proteins. Multiple functional proteins, such as the tumor suppressor proteins p53, RB, PTEN, upon stabilization by DUBTAC, can effectively restore or enhance their physiological functions, thus achieving therapeutic effects. Currently, the DUBTAC technology is still in its early stage of development, yet it has broad application prospects and represents a technological approach for developing various “undruggable” targets. This article delves into the design strategy of DUBTAC, and screens and recommends some candidate proteins with the potential to serve as drug targets. We aim to provide perspective in drug design, structural optimization, target selection, and related aspects.

## Graphical abstract

PROTACs and DUBTACs have emerged as two crucial paradigms for regulating protein homeostasis, offering novel therapeutic approaches for complex diseases.

**Figure:**
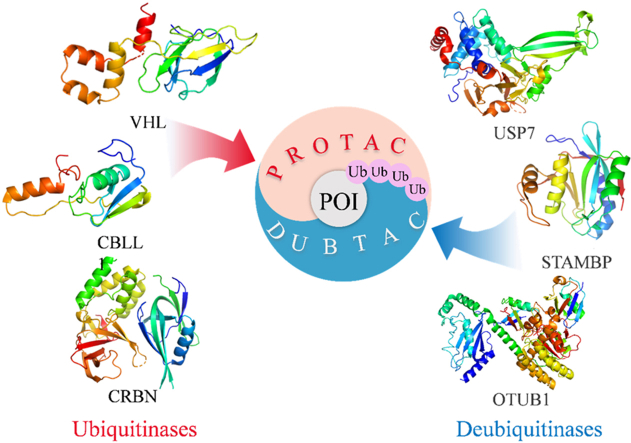

## 1 Introduction

Inside the cell, the ubiquitination and deubiquitination of proteins are in a delicate dynamic balance, in which deubiquitinases (DUBs) play an indispensable role. DUBs cleave ubiquitin from substrate proteins, allowing them to function continuously and stably^1^. This leads researchers to realize that DUBs can be harnessed to influence protein stability, thus uncovering novel pathways for drug development. In order to precisely utilize DUBs to regulate protein stability, the design concept of deubiquitinase-targeting chimeras (DUBTAC) has emerged. DUBTAC can recruit DUBs to specific target proteins, thereby achieving deubiquitination of the target proteins and regulating their stability. This targeted strategy offers a novel approach to addressing the challenges of protein stability regulation in cancer treatment^2^.

**Figure 1:**
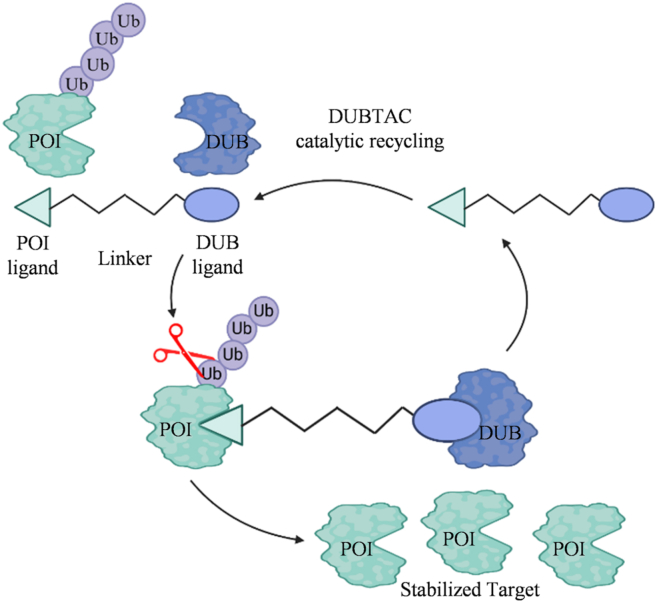
DUBTAC-driven protein stabilization. DUBTACs are heterobifunctional compounds composed of a protein–targeting ligand conjugated to a DUB recruiter through a chemical linker. Upon cellular application, DUBTACs facilitate proximity between a DUB and the target protein, enabling removal of polyubiquitin chains. This process inhibits proteasome-mediated degradation, thereby stabilizing and increasing cellular levels of the otherwise rapidly degraded protein.

DUBTAC is a class of artificially designed and synthesized bifunctional molecules. One end of a DUBTAC molecule is linked to a ligand that can specifically bind to the target protein, while the other end is connected to a deubiquitinase^3^. The fundamental principle is that one ligand precisely anchors the target protein, bringing the target protein and the DUBs closer together, and subsequently removing the ubiquitin chains that have been attached to the target protein^4^. Once the DUBs remove the ubiquitin chains, the target protein can “escape” the fate of degradation, maintaining a stable level, and in some cases, even having its function restored. This technology has the ability to stabilize some key functional proteins, enabling them to exert a more sustained and effective impact (Fig. 1)^5^.

Most traditional small molecule drugs exert their effects by inhibiting protein activity^6^. However, when it comes to some diseases caused by the loss of protein function, stabilizers or agonists are the ones that most need to be developed. DUBTAC takes an opposite approach, focusing on stabilizing specific proteins and maintaining the concentration of functional proteins. In some research on neurodegenerative diseases, such as Parkinson’s disease, the pathogenic mechanisms include the depletion of certain neuroprotective proteins^7^^,^^8^. DUBTAC can target these proteins, prevent their excessive ubiquitination and degradation, maintain the homeostasis of proteins in the brain, alleviate neuronal damage, and holds promise in breaking the current treatment impasse. In oncological therapeutics, a critical pathogenic mechanism involves tumor cells exploiting the ubiquitin‒proteasome system (UPS) to drive dysregulated degradation of tumor suppressor proteins such as p53, thereby facilitating malignant proliferation and metastatic dissemination^9^. Counteracting this oncogenic mechanism, the DUBTAC platform emerges as a precision therapeutic approach capable of restoring tumor-suppressive functions through dual molecular interventions: targeted stabilization of tumor suppressor proteins and re-establishment of proliferative homeostasis, ultimately achieving potent suppression of tumorigenic progression^10^^,^^11^.

DUBTAC has expanded the boundaries of drug development, inspiring scientists to design innovative therapies based on the regulation of protein homeostasis. Compared with the complexity and potential risks of gene therapy, DUBTAC by virtue of its characteristics, exhibits better cell penetration and pharmacokinetic properties. It is more amenable to drug development, and its cost is more controllable.

## 2 Differences between DUBTAC and PROTAC

**Figure 2:**
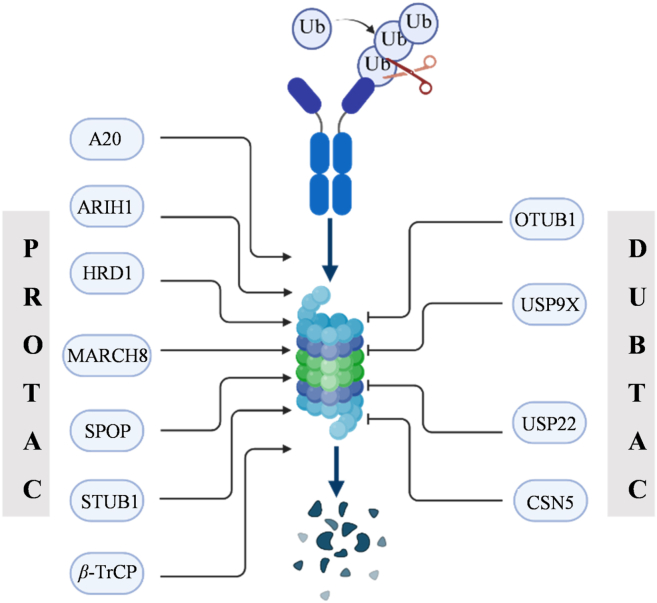
Protein degradation is controlled by a network of ubiquitin ligases and deubiquitinating enzymes. Therapeutic selection between PROTAC and DUBTAC technologies depends on disease pathophysiology. In cancer, PROTACs recruit E3 ligases (*β*-TrCP/SPOP/STUB1 et al.) to induce PD-L1 degradation *via* the ubiquitin-proteasome system, enhancing immune activation. Conversely, in autoimmune disorders, DUBTACs engage DUBs (CSN5/USP22/OTUB1 et al.) to stabilize PD-L1 through ubiquitin chain excision, thereby preventing immune hyperactivation.

In terms of the mechanism of action, the core of proteolysis targeting chimera (PROTAC) technology lies in ubiquitination driven degradation. One end of PROTAC binds to the target protein, and the other end docks with the E3 ligase. As a key enzyme in the ubiquitination process, the E3 ligase covalently attaches ubiquitin molecules to the target protein^12^^,^^13^. When PROTAC brings the two entities into close proximity and facilitates their binding, the target protein undergoes continuous ubiquitination, thereby forming a polyubiquitin chain^14^. This “tag” is recognized by the proteasome, ultimately leading to the capture and degradation of the target protein by the 26S proteasome^15^. In contrast, DUBTAC aims to rescue the target protein. It also consists of two parts: one end is a ligand that can specifically recognize the target protein, and the other end is linked to a DUB. After entering the cell, the ligand quickly locates and tightly binds to the target protein, bringing the DUBs close and removing the ubiquitin chains already attached to the target protein. The target protein, having lost its “degradation tag”, can escape the “capture” of the proteasome, maintaining a stable protein level and function. This provides a novel strategy for treating diseases caused by excessive protein degradation (Fig. 2)^3^.

In conclusion, the DUBTAC and PROTAC platforms exhibit complementary therapeutic benefits. DUBTAC stabilizes target proteins by means of deubiquitination mechanisms, whereas PROTAC drives proteasomal degradation through ubiquitination pathways. The strategic choice between these two protein homeostasis modulation strategies, which is guided by target specific features such as pathological function (oncogenic *versus* tumor-suppressive), subcellular localization, and the context of the disease microenvironment, allows for the maximization of therapeutic efficacy in precision medicine applications.

## 3 Warheads targeting DUBs

**Figure 3:**
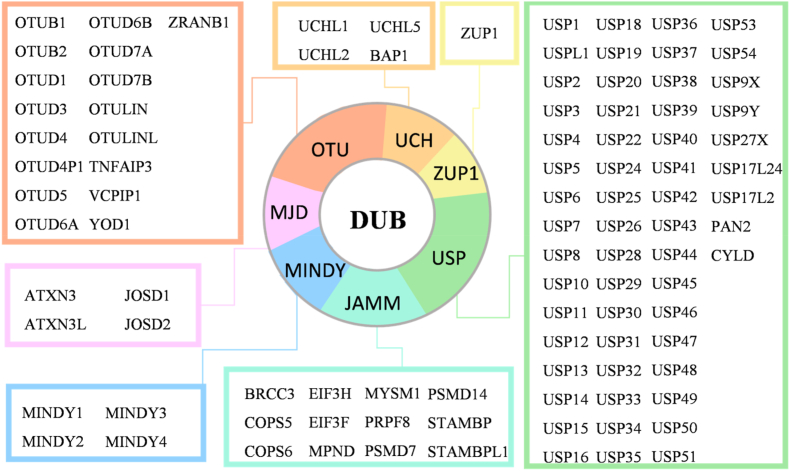
The list of human deubiquitinating enzymes. Approximately 100 human deubiquitinating enzymes are categorized into seven families based on catalytic domain sequence homology: ubiquitin-specific proteases (USP), ubiquitin C-terminal hydrolases (UCH), Machado-Josephin domain proteases (MJD), Jab1/Mov34/Mpr1 Pad1 N-terminal + domain proteases (JAMM), ovarian tumor proteases (OTU), and zinc finger-containing ubiquitin peptidase 1 (ZUP1).

Seven classes of DUBs have been discovered, including 56 ubiquitin-specific peptidases (USPs), 12 Jab1/Mov34/Mpr1 Pad1 N-terminal + domain proteases (JAMMs), 17 ovarian tumor proteases (OTUs), 4 Machado-Josephin domain proteases (MJDs), 4 ubiquitin C-terminal hydrolases (UCHs), 5 motif interacting with ubiquitin-containing novel DUB family proteases (MINDYs), and zinc finger-containing ubiquitin peptidase 1 (ZUP1)^16^. These deubiquitinating enzymes catalyze the hydrolysis of isopeptide bonds linking ubiquitin moieties to substrate proteins, as well as peptide bonds within ubiquitin precursor polypeptides (Fig. 3). Through these dual catalytic activities, they precisely remove ubiquitin molecules from either polyubiquitinated substrates or pro-ubiquitin precursors, thereby executing essential roles in ubiquitin chain editing, substrate rescue, and ubiquitin recycling during proteostasis regulation^17^. As critical counterregulatory components of the UPS, DUBs dynamically modulate protein stability through precise editing of ubiquitin chains, thereby exerting spatiotemporal control over diverse cellular processes including signal transduction, DNA repair, and apoptosis^1^^,^^18^. Moreover, the warheads–DUB–ligands that can precisely target DUBs have become the forefront of chemical biology research. Existing DUB ligands have already shown their unique values, and the development of novel ligands is also progressing steadily (Table 1)^19^, ^20^, ^21^, ^22^, ^23^, ^24^, ^25^, ^26^, ^27^, ^28^, ^29^, ^30^, ^31^, ^32^, ^33^, ^34^, ^35^, ^36^, ^37^, ^38^, ^39^^,^^40^, ^41^, ^42^, ^43^, ^44^, ^45^, ^46^, ^47^, ^48^, ^49^, ^50^, ^51^, ^52^, ^53^, ^54^, ^55^, ^56^, ^57^, ^58^, ^59^, ^60^, ^61^, ^62^, ^63^.

**Table 1:** Target proteins and small molecule ligands of DUBs.

Target	Functional protein	Molecule structure	Binding pocket	Ref.
OTUB1	p53, AKT, GRAIL, MDMX, SOCS1, FOXM1		Non-catalytic pocket	19, 20, 21, 22
			Non-catalytic pocket	
USP1	PCNA, ub-FANCD2, ub-FANCI		Non-catalytic pocket	23, 24, 25, 26
			Non-catalytic pocket	
USP4	p53, TGF*β*-I, Cyclin A1, RNPS1, SMAD4		Non-catalytic pocket	27, 28, 29
			Not defined	
USP5	CCND1, STAT3, TRAF6, RIG-I		Not defined	30, 31, 32, 33, 34
			Not defined	
			Not defined	
USP7	p53, MDM2, MDMX, FOXO4, PTEN, Cry1, CHFR, Chk1		Non-catalytic pocket	35, 36, 37, 38
			Non-catalytic pocket	
			Non-catalytic pocket	
USP8	CHMP, p62, Parkin		Not defined	39, 40, 41, 42
USP10	p53, CFTR, AMPK*α*, SIRT6, PTEN,		Not defined	43, 44, 45
USP14	p53, PTEN		Non-catalytic pocket	46,47
			Non-catalytic pocket	
USP30	Parkin, PEX5, ACLY		Not defined	48, 49, 50, 51
			Not defined	
			Not defined	
UCHL1	TrkB, HIF1*α*, BACE1		Non-catalytic pocket	52, 53, 54, 55, 56
PSMD14	E2F1, GRB2, TGFBR1, ALK2		Non-catalytic pocket	57, 58, 59, 60, 61
			Not defined	
			Not defined	
JOSD2	LKB1, PHGDH, PFK-1, SERCA2a		Not defined	62,63

However, not all DUB-binding partners are suitable as recruiting ligands for DUBTACs. Ideal ligands should act as agonists or bind to non-catalytic regions. For instance, EN523 targets an allosteric site on OTUB1, facilitating non-competitive inhibition while preserving deubiquitinating activity within a DUBTAC complex. In contrast, small-molecule inhibitors directed at the catalytic pocket are theoretically poor candidates for DUBTAC design. Nevertheless, existing experimental evidence demonstrates that inhibitory ligands, such as the USP1-directed compound **55**, can still promote target protein stabilization and exhibit therapeutic efficacy across multiple disease models. This suggests that even partially inhibited DUBs may retain adequate catalytic function to mediate productive deubiquitination. The mechanisms underlying this apparent contradiction, however, remain unclear and lack systematic investigation. We anticipate that further mechanistic studies into the functional phenotypes, binding kinetics, and spatial modes of action of DUB ligands will help reconcile theoretical predictions with empirical observations, thereby advancing DUBTAC technology from empirical discovery toward rational and precise design.

Within the seven major classes of DUBs, each subclass orchestrates distinct regulatory nodes in the UPS through specialized substrate recognition and catalytic mechanisms^64^. Of particular biomedical significance, OTU domain-containing ubiquitin aldehyde-binding protein 1 (OTUB1) is a DUB encoded by the *OTUB1* gene. Its protein structure features a typical ovarian tumor domain, which enables it to specifically recognize and cleave ubiquitin chains, thereby reversing the ubiquitination modification of proteins^65^. For instance, OTUB1 can deubiquitinate and stabilize proteins, regulate the activity and function of p53, and influence processes such as the growth and apoptosis of tumor cells^66^. In 2022, during the design of DUBTACs, the Nomura research group^3^ developed EN523, a small-molecule covalent ligand that targets the novel deubiquitinase OTUB1. Building upon the foundation laid by previous research, in 2024, a research team spearheaded by Professor Jian Jin from the Icahn School of Medicine at Mount Sinai and Professor Wenyi Wei from Harvard Medical School^4^ embarked on the optimization of EN523. Their efforts culminated in the successful development of a novel OTUB1 ligand, MS5105. Significantly, MS5105 not only further elevates the efficiency of covalently modifying OTUB1 but also, as a direct consequence of this enhancement, renders the design of highly efficient DUBTACs more feasible and promising.

Ubiquitin-specific protease 7 (USP7) is an important DUB with multiple functional domain^35^. Its catalytic domain is highly conserved and serves as the core region for exerting DUBs activity. It can specifically recognize and cleave the isopeptide bond between the ubiquitin chain and the target protein, thereby regulating the stability and function of the target protein^67^. For instance, USP7 deubiquitinates AMP-activated protein kinase (AMPK), significantly enhancing the stability of AMPK and ensuring that AMPK continuously exerts its metabolic regulatory function, thus maintaining the energy homeostasis of cells. In recent years, remarkable progress has been made in developing small-molecule inhibitors targeting USP7, such as USP7 ligand #1, USP7i-#4a, and GNE6776. These small molecules can serve as effective ligands for USP7 DUBTACs, remarkably stabilizing certain key proteins, such as cystic fibrosis transmembrane conductance regulator (CFTR) and AMPK^5^.

Ubiquitin-specific protease 10 (USP10) belongs to the USP family and plays a central role in cellular metabolism, signal transduction, and tumor suppression. Studies have shown that USP10 is activated under cellular stress conditions (*e.g.*, oxidative stress or DNA damage), where it stabilizes key proteins through deubiquitination to enable cellular stress adaptation^43^. For example, USP10 stabilizes p53 *via* deubiquitination and enhances its transcriptional activity, thereby contributing to DNA damage repair and apoptosis regulation^45^. Additionally, USP10 regulates autophagy-related proteins. For instance, it deubiquitinates Beclin-1, promoting autophagosomes formation and autophagy activation^44^. Currently, small molecule inhibitors targeting USP10, such as HBX19818, are in preclinical development, with no highly specific modulators reported to date^68^.

STAM-binding protein (STAMBP), alternatively designated associated molecule with the SH3 domain of STAM (AMSH), is a DUB linked to the endosomal sorting complex required for transport (ESCRT)^69^. Belonging to the JAMM/MPN + family, STAMBP is predominantly enriched on the endosomal membrane where it regulates the endocytosis and sorting processes of cell surface receptors. It governs the stability of receptor proteins and their intracellular trafficking *via* deubiquitination modification^70^. For instance, nucleotide binding oligomerization domain like receptor protein 3 (NLRP3), a crucial protein in the TLR signaling pathway, which is part of the immune signaling pathway, serves as one of the targets of STAMBP. During pathogen invasion, STAMBP precisely modulates NLRP3 ubiquitination status to balance immune responses, preventing both hyperactivation and insufficient signaling^71^. Furthermore, STAMBP is also engaged in regulating the sorting and degradation of other receptor proteins, such as C–X–C chemokine receptor type 4 (CXCR4), a key regulator of cell migration and immune responses^72^. Despite its therapeutic potential in cancer biology, small-molecule inhibitors targeting STAMBP remain largely experimental, with no clinically validated agents reported to date.

Ubiquitin C-terminal hydrolase L1 (UCHL1), a neuron-specific deubiquitinase belonging to UCH family^73^. It is abundantly expressed in the central nervous system and germ cells, constituting approximately 5% of total neuronal protein content. This enzyme is linked to neurodegenerative diseases as well as germ cell development^54^^,^^73^. Additionally, UCHL1 regulates critical processes including spermatogenesis, oocyte maturation, and fertilization. A pivotal aspect of UCHL1 function involves its interaction with *α*-synuclein, a protein implicated in Parkinson’s disease (PD) pathogenesis. By modulating *α*-synuclein ubiquitination status, UCHL1 influences its aggregation propensity a hallmark of PD neuropathology^74^. Through the STRING database, UCHL1 has been recognized as a binding partner of both *α*-synuclein and PD 2^55^. Current research on UCHL1 modulation primarily focuses on small-molecule inhibitors such as LDN-57444 and LDN-91946, which have demonstrated efficacy in functional studies^56^. However, agonists of UCHL1 have not been reported, and its potential as a therapeutic target for neurodegenerative diseases still needs further exploration.

In conclusion, DUBs regulate protein homeostasis by modulating ubiquitination, a critical post-translational modification. Pathological conditions characterized by aberrant ubiquitination patterns create therapeutic opportunities, as these misregulated proteins become prime targets for DUBTAC technologies. However, DUBs present considerable challenges as therapeutic targets. Owing to issues such as low selectivity and potential toxicity, most small-molecule DUB inhibitors have failed to progress into clinical use, with very few exceptions. Directly repurposing such inhibitors for DUBTAC construction would inherently carry over their off-target effects and systemic toxicities, greatly limiting clinical translation. Thus, in designing DUBTACs, priority should be given to DUB ligands with existing clinical data and established safety profiles to improve developability. Early integration of system-wide approaches, such as chemoproteomics and ubiquitomics, is crucial to evaluate systematically the effects of candidate molecules on global DUBs activity and ubiquitin signaling, facilitating early risk mitigation and prioritization of non-inhibitory, allosteric, and highly selective binders. Although the current repertoire of validated DUB ligands remains limited and no consensus “ideal” ligand has yet emerged, advances in mechanistic insight may eventually focus efforts on a subset of DUBs with favorable safety and functional profiles. This progression will support the evolution of DUBTAC technology from broad discovery toward targeted and rationally designed therapeutics.

## 4 Target proteins and small-molecule ligands of DUBTAC

**Figure 4:**
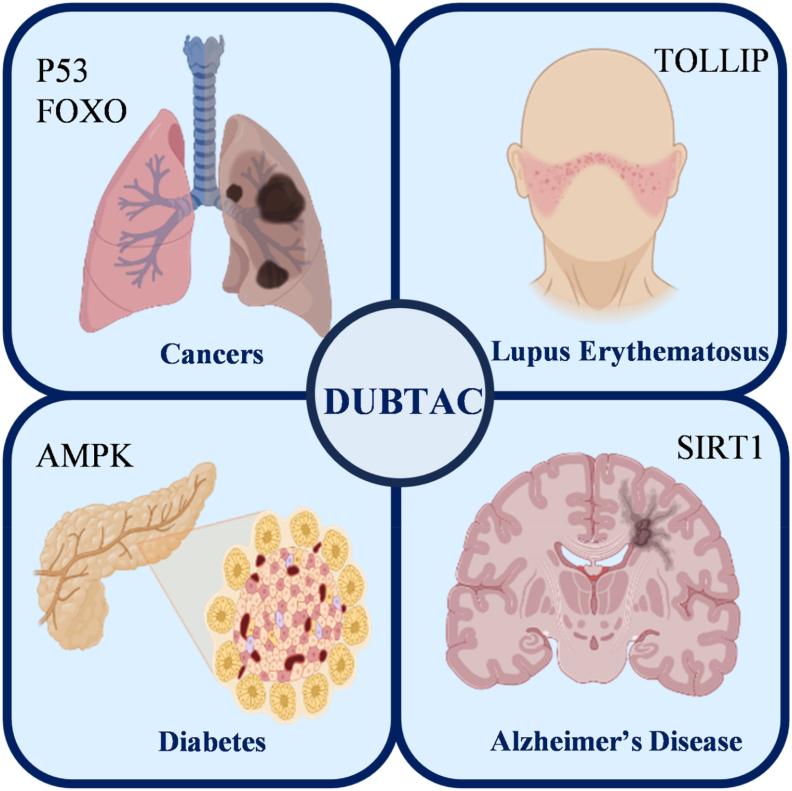
DUBTAC is applicable to a range of pathological conditions. DUBTACs represent a novel therapeutic modality for refractory diseases, employing a modular architecture with broad-spectrum applicability across oncology, autoimmune disorders, infectious pathologies, cardiovascular conditions, and neurodegenerative processes.

**Figure 5:**
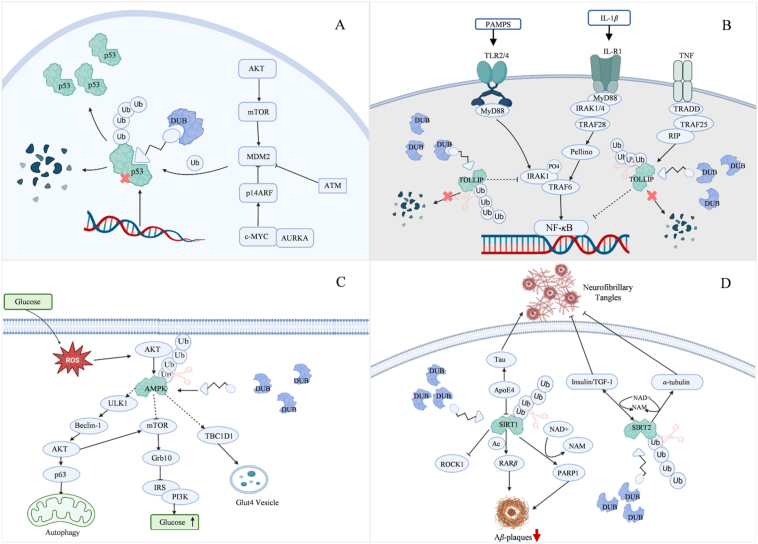
Mechanisms of DUBTACs in Disease Pathology. (A) In cancer, MDM2 overexpression promotes p53 degradation. p53–DUBTAC recruits DUBs to stabilize p53, slowing tumor progression. (B) In autoimmune diseases, TNF-*α*-induced TOLLIP degradation leads to NF-*κ*B overactivation. TOLLIP–DUBTAC stabilizes TOLLIP, restoring NF-*κ*B regulation. (C) In type 2 diabetes, high glucose levels promote the generation of reactive oxygen species (ROS) in cells, which activate AKT. As a result, AMPK is ubiquitinated and fails to normally activate pathways such as mTOR/ULK1/TBC1D1 to reduce blood glucose levels. AMPK–DUBTACs can recruit deubiquitinating enzymes to remove ubiquitin, stabilize the protein, and slow down the progression of the disease. (D) In neurodegenerative diseases, the aggregation of Tau protein can promote mitochondrial dysfunction and reduce the expression of SIRT1/2, facilitating the formation of neurofibrillary tangles. SIRT1/2–DUBTACs can recruit deubiquitinating enzymes to remove ubiquitin, stabilize the protein, and enable it to function properly.

DUBTACs are emerging as transformative therapeutic platforms in modern biomedical research, offering novel intervention strategies for intractable pathologies. This modular technology demonstrates broad-spectrum therapeutic potential, with applications encompassing oncology, autoimmune disorders, infectious diseases, cardiovascular pathologies, and neurodegenerative conditions (Fig. 4). Elucidating the structural and conformational dynamics of target proteins, their pathogenic mechanisms, and the regulatory interplay with cognate DUBs will be instrumental for advancing next-generation precision therapies (Fig. 5). These insights are driving scientific paradigms in drug discovery, providing foundational frameworks to address complex disease.

In cancer biology, the p53 tumor suppressor serves as a master regulator governing genomic integrity maintenance, cell cycle checkpoint control, and apoptosis induction^11^. Mechanistically, under genotoxic stress induced by ultraviolet radiation or chemical carcinogens, DNA damage sensors ATM/ATR kinase initiate p53 activation through phosphorylation of specific serine residues (*e.g.*, Ser15 and Ser20), thereby stabilizing and allosterically activating p53 by disrupting its MDM2-mediated ubiquitination^75^^,^^76^. This post-translational modification enables nuclear p53 to function as a sequence-specific transcription factor, directing transcriptional programs that determine cell fate^77^. Upon irreparable DNA damage, p53 transactivates pro-apoptotic effectors such as BAX^78^. Hich oligomerizes to permeabilize mitochondrial outer membranes. This mitochondrial permeabilization initiates cytochrome release, subsequently activating the caspase cascade and eliminating genomically compromised cells^11^.

In cancers such as colorectal, lung, hepatocellular, prostate, and cutaneous cancer, p53 protein levels are frequently downregulated, leading to the impairment of its tumor suppressor function. This deficiency enables cancer cells to evade the regulation of the cell cycle and the apoptosis pathways, facilitating uncontrolled proliferation and metastasis. Current research efforts have developed multiple p53 agonists. Notable examples include alrizomadlin, eprenetapopt, and CBL-0137, which are currently in Phase II clinical trials. These small-molecule agents function as targeted therapies to reactivate p53^79^, ^80^, ^81^. Regarding deubiquitination regulation, p53 stability is controlled by multiple DUBs, including USP7, OTUB1, and USP10. USP7 and OTUB1 bind to the MDM2‒p53 complex, cleaving ubiquitin chains from MDM2 to modulate p53 ubiquitination^82^^,^^83^. Lou Zhenkun’s team^84^ identified USP10 as a p53-specific DUBs through co-immunoprecipitation and gene silencing assays. Functional studies demonstrate that USP10 overexpression in clear cell carcinoma cells can effectively suppress tumorigenicity. Existing DUB inhibitors targeting USP7/OTUB1 have been developed^3^^,^^4^. When paired with p53 agonists, these ligands can form bifunctional DUBTACs to enhance therapeutic efficacy.

Beyond p53, the Forkhead box O (FOXO) transcription factor has emerged as a pivotal therapeutic target in oncology^85^. This protein regulates diverse biological processes including cellular development, metabolism, stem cell homeostasis, and lifespan regulation^86^^,^^87^. Notably, FOXO proteins activate pro-apoptotic Bcl-2 family members such as Bcl-2 interacting mediator of cell death (Bim), fatty acid synthase-ligand (Fasl), and TNF-related apoptosis-inducing ligand (TRAIL), which induce programmed cell death in both normal and malignant cells^88^. At the transcriptional level, FOXO1 enhances T-cell trafficking through CCR7 upregulation while modulating CD8^+^ T-cell differentiation *via* T-bet transcriptional repression^89^. In cancer context, FOXO downregulation promotes tumorigenesis through dual mechanisms: apoptosis evasion and metabolic dysregulation^90^. The DUBTAC strategy addresses this deficiency by stabilizing FOXO proteins to restore their tumor-suppressive functions. While no FOXO-specific DUBs have been characterized, recent advances utilize DNA oligonucleotide (ODN)-based TF-DUBTAC platforms to maintain FOXO stability through targeted deubiquitination^91^. Despite these innovations, small-molecule FOXO agonists remain underexplored, with no clinically approved agents targeting this pathway to date.

In autoimmune pathologies such as rheumatoid arthritis (RA) and systemic lupus erythematosus (SLE), Toll-interacting protein (TOLLIP) emerges as a critical immune regulator^92^^,^^93^. The TOLLIP protein predominantly participates in the negative regulation of the Toll-like receptor (TLR) signaling pathway^94^^,^^95^. TLRs function as pattern recognition receptors (PRRs) that detect pathogen-associated molecular patterns (PAMPs) and damage-associated molecular patterns (DAMPs), and they play a vital part in initiating the innate immune response^96^. Upon activation, TLRs recruit a cascade of adapter proteins, including myeloid differentiation factor 88 (MyD88), to initiate the downstream signaling cascade. This ultimately leads to the activation of transcription factors like nuclear factor-*κ*B (NF-*κ*B) and stimulates the expression of inflammatory cytokines^97^^,^^98^. Dysregulated TLR signaling in autoimmune diseases leads to loss of self-tolerance through aberrant cytokine release and autoreactive immune cell activation^99^. TOLLIP regulates immune homeostasis through dual mechanisms. The TOLLIP protein engages with adaptor proteins including MyD88 to suppress downstream signaling cascades, thereby modulating TLR pathway activity and preventing immune hyperactivation^92^^,^^100^^,^^101^. TOLLIP maintains STING stability in its inactive state by binding to the protein and preventing lysosomal degradation, thereby preserving immune homeostasis^102^.

In rheumatoid arthritis, TOLLIP deficiency impairs its capacity to suppress aberrant inflammatory activation^93^. This dysregulation leads to sustained synovial immune cell hyperactivity^103^^,^^104^, driving pathological cytokine cascades (tumour necrosis factor-*α*, interleukin-6) that promote synovial hyperplasia, pannus formation, and articular destruction, culminating in joint dysfunction^105^. Current therapeutic targeting of TOLLIP remains experimental, with no validated DUB regulators identified. Notably, USP18 demonstrates preferential expression in primary immune tissues (spleen, lymph nodes, thymus)^106^, ^107^, ^108^. Targeted DUBTAC design coupling USP18 recruitment to TOLLIP could enhance proteostasis of this immunomodulator, presenting a novel therapeutic avenue for autoimmune pathologies including systemic lupus erythematosus. These mechanistic insights expand DUBTAC’s therapeutic potential beyond single targets, enabling precision modulation of complex immune networks.

Cardiovascular diseases constitute a leading global health challenge, imposing significant morbidity and mortality burdens worldwide^109^. Within the cardiovascular system’s sophisticated regulatory framework, AMPK serves as a master regulator of metabolic homeostasis^110^. As a conserved energy sensor, AMPK dynamically monitors cellular energy status through real-time detection of AMP/ATP ratio fluctuations^111^. During myocardial ischemia, energy depletion triggers AMP accumulation, which induces conformational changes in AMPK’s *γ*-subunit, activating its kinase domain^112^. This enzymatic activation initiates coordinated catabolic and anabolic responses: stimulating mitochondrial fatty acid oxidation to meet myocardial energy demands, suppressing lipogenic pathways to prevent lipid overload, and modulating endothelial nitric oxide synthase (eNOS) activity to enhance vascular perfusion^113^, ^114^, ^115^. These pleiotropic effects position AMPK as a critical therapeutic node for preserving cardiac energetics while maintaining vascular homeostasis.

Pathological conditions such as hypertension and dyslipidemia induce sustained AMPK signaling dysfunction through chronic metabolic stress^116^. AMPK protein deficiency triggers pleiotropic metabolic disturbances, including impaired glucose homeostasis, diminished fatty acid oxidation capacity, and marked insulin resistance, culminating in hyperglycemia^117^, ^118^, ^119^. Current AMPK-targeted pharmacological strategies include US Food and Drug Administration-approved metformin, which enhances AMPK activation *via* liver kinase B1 (LKB1) phosphorylation^120^^,^^121^, alongside clinical-stage molecules ATX-304, PXL-770, and Antroquinonol^122^, ^123^, ^124^, ^125^. Emerging regulatory paradigms reveal USP10-mediated deubiquitination stabilizes AMPK through K48-linked polyubiquitin chain removal^45^. Pioneering this field, Professor Wenyi Wei’s team^5^ at Harvard Medical School recently developed a DUBTAC-based bifunctional molecule that recruits USP10 to AMPK *via* a protease-cleavable linker system. This innovative platform achieves AMPK stabilization *in vitro*, overcoming traditional agonist limitations while maintaining spatial regulation.

Sarco/endoplasmic reticulum calcium ATPase 2a (SERCA2a) constitutes a key membrane transporter in cardiovascular pathophysiology, orchestrating intracellular calcium homeostasis through ATP-dependent sarcoplasmic reticulum (SR) calcium sequestration^126^. This SR-resident pump utilizes ATP hydrolysis to translocate cytosolic calcium against its concentration gradient during myocardial diastole^127^, reducing cytoplasmic calcium levels essential for myocardial relaxation and diastolic filling^128^^,^^129^. Pathological downregulation or functional impairment of SERCA2a observed in hypertension, myocardial infarction^130^, and cardiomyopathy disrupts calcium homeostasis, leading to cytosolic calcium overload^131^. This dysregulation triggers dual pathogenic cascades: sustained cytosolic calcium elevation both increases myocardial contractile force through enhanced myofilament sensitivity, driving cardiomyocyte hypertrophy^132^, and activates calcineurin/NFAT and MAPK signaling pathways that promote pathological remodeling^133^. The resulting ventricular remodeling manifests as myocardial hypertrophy with chamber stiffening, fibrotic matrix deposition, and progressive ventricular dysfunction that culminates in heart failure^134^. Current development of small-molecule SERCA2a agonists remains challenging, creating a critical therapeutic gap that underscores the strategic value of DUBTAC technology. USP25 and Josephin domain containing 2 (JOSD2) regulate SERCA2a stability through deubiquitination mechanisms, stabilizing this calcium transporter to maintain cardiomyocyte calcium homeostasis and myocardial function^63^^,^^135^. Despite these physiological regulatory mechanisms, no clinical-stage small-molecule modulators targeting the SERCA2a–USP25–JOSD2 axis have been reported. However, DUBTAC-based modulation represents a promising therapeutic strategy that remains underexplored for this system.

In the context of neurodegenerative disorders, the silent information regulator (SIRT) family of proteins represents a critical area of neurological research^136^. Comprising seven isoforms (SIRT1‒SIRT7), these nicotinamide adenine dinucleotide (NAD^+^)-dependent deacetylases regulate pivotal biological processes including cellular senescence, survival mechanisms, and neuroprotection within the nervous system^137^. A paradigmatic example is SIRT1, which modulates transcriptional activity and cellular homeostasis through deacetylation of key regulatory proteins^138^. Mechanistically, SIRT1 deacetylates p53 to suppress apoptotic pathways, thereby preserving neuronal integrity^139^^,^^140^. Concurrently, its deacetylation of FOXO transcription factors enhances antioxidant gene expression, reinforcing cellular defenses against oxidative stress^141^. Preclinical studies corroborate SIRT1’s neuroprotective role^142^, demonstrating that its upregulation improves cognitive function while delaying the onset and progression of neurodegenerative pathologies^143^. Notably, activation of the AMPK/SIRT1 signaling axis has shown therapeutic potential in models of Alzheimer’s disease (AD), Parkinson’s disease, and postoperative cognitive dysfunction (POCD), effectively mitigating cognitive deficits through pleiotropic protective mechanisms^144^.

With aging or under specific pathological conditions, cellular NAD^+^ levels decline, leading to reduced SIRT1 activity and expression^145^. SIRT1 dysfunction disrupts neuronal protein homeostasis, exacerbates oxidative stress, and accelerates pathologic processes including apoptosis^146^^,^^147^. Current clinical development of SIRT-targeting therapies includes several small-molecule agonists such as resveratrol and selisistat^148^^,^^149^. As one of the earliest identified SIRT1 activators, resveratrol enhances enzymatic deacetylation through direct protein binding^150^. These agonists may serve as molecular scaffolds for developing SIRT-focused DUBTACs. Although specific DUB partners for SIRTs remain undefined, USP8, OTUB1, and UCH-L1 all implicated in Alzheimer’s disease pathogenesis exhibit neuronal expression^151^, ^152^, ^153^. Leveraging validated DUB ligands from these studies could enable rational design of novel SIRT–DUBTAC platforms, potentially pioneering innovative therapeutic strategies for neurodegeneration.

DUBTAC technology has emerged as a transformative therapeutic platform with multi-disease targeting capabilities, showcasing unprecedented therapeutic versatility across oncology, autoimmune disorders, cardiovascular pathologies, and neurodegenerative diseases. However, a major challenge lies in the absence of known ligands for many target proteins, which severely restricts the design and development of DUBTACs. Therefore, when selecting suitable targets, priority should be given to whether pathological hyper-ubiquitination is present, as this criterion provides critical guidance for subsequent research. In recent years, advancements in computational power and algorithms have established artificial intelligence (AI) as a powerful tool for accelerating drug discovery. In the PROTAC field, machine learning models have been employed to predict compound–target binding affinity, optimize linker design, and assess pharmacokinetic properties. For example, the Bai group^154^ developed DiffPROTACs, a diffusion-based generative model that integrates diffusion models with Transformer algorithms. By leveraging the Transformer to learn and generate novel linkers compatible with given ligands, DiffPROTACs achieves a validity rate of 93.86% and structural uniqueness of 68.7%. These AI technologies are equally applicable to DUBTAC development and hold promise for significantly accelerating the process of molecular design and optimization.

## 5 Specific implementation case

**Figure 6:**
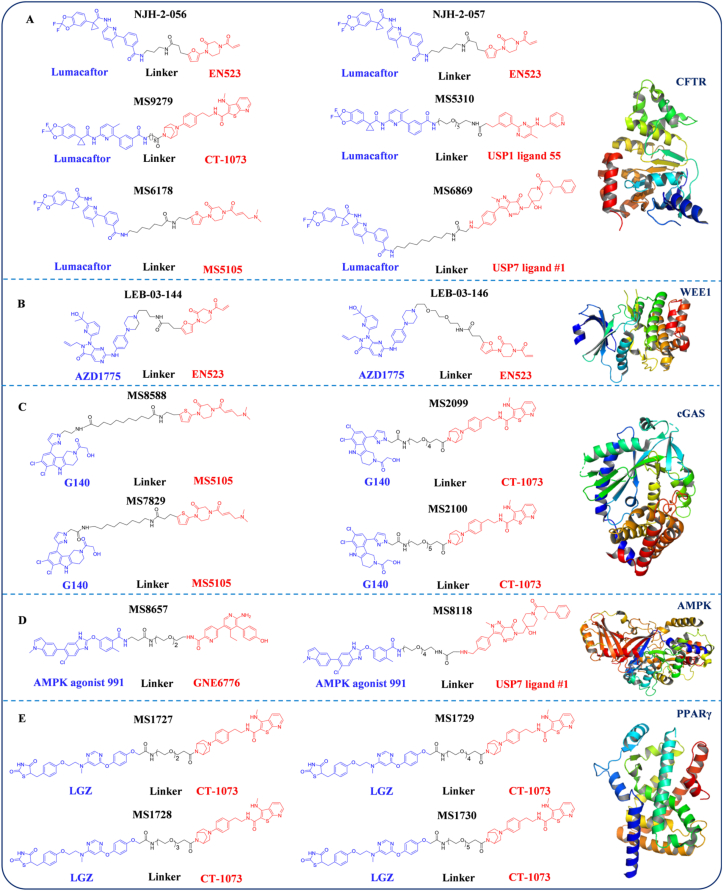
Co-crystal structures of target proteins with existing DUBTAC bifunctional molecules. (A) The CFTR protein (PDB:4WZ6) and its CFTR–DUBTACs: NJH-2-056, NJH-2-057, MS9279, MS5310, MS6178 and MS6869. (B) The WEE1 protein (PDB:5V5Y) and its WEE1–DUBTACs: LEB-03-144 and LEB-03-146. (C) The cGAS protein (PDB:6CTA) and its cGAS–DUBTACs: MS8588, MS7829, MS2099, and MS2100. (D) The AMPK protein (PDB:4CFF) and its AMPK–DUBTACs: MS8657 and MS8118. (E) The PPAR*γ* protein (PDB:3AN4) and its PPAR*γ*–DUBTACs: MS1727‒MS1730.

DUBTACs represent a paradigm-shifting proteostasis engineering platform through bifunctional molecules that recruit DUBs to disease-associated substrates. This modular design enhances target protein stability *via* site-specific deubiquitination while restoring physiological proteostasis. Emerging preclinical evidence validates therapeutic efficacy in refractory protein-misfolding disorders, exemplified by CFTR rescue in cystic fibrosis (ΔF508-CFTR folding correction) and insulin signaling restoration in type 2 diabetes (Fig. 6). These proof-of-concept studies demonstrate DUBTACs' unique capacity to pharmacologically modulate previously undruggable proteostatic nodes, establishing this platform as a cornerstone for targeted therapies against ubiquitination-dependent pathologies.

Cystic fibrosis (CF) is an autosomal recessive disorder caused by mutations in the *CFTR* gene^155^. The ΔF508 mutation—resulting from codon 508 deletion in exon 10—represents the most prevalent pathogenic variant, causing phenylalanine loss that destabilizes CFTR conformation^156^. This structural instability triggers K48-linked polyubiquitination, leading to premature proteasomal degradation before CFTR reaches the plasma membrane^157^. The resulting chloride channel dysfunction disrupts ion/fluid homeostasis in respiratory epithelia, creating a pro-inflammatory milieu that promotes bacterial colonization and disease progression^158^. The US Food and Drug Administration-approved pharmacological chaperone Lumacaftor partially rescues ΔF508-CFTR folding, enhancing membrane localization and functional recovery^159^. However, despite this corrective effect, most mutant proteins remain susceptible to rapid ubiquitination and proteasomal degradation within the secretory pathway^160^. This therapeutic limitation underscores the need for novel strategies capable of stabilizing misfolded CFTR. DUBTAC technology now offers a potential solution by combining deubiquitinating enzyme activation with conformational stabilization, presenting a precision approach to address both quality control defects and functional deficits in CFTR biology.

In 2022, Nomura and colleagues^3^ demonstrated that the Cys23 residue in deubiquitinase OTUB1 plays no role in enzymatic regulation while exhibiting high nucleophilicity, making it an ideal ligand-binding site. Leveraging these findings, they subsequently identified a covalent ligand, EN523, which binds OTUB1 with high affinity through its acrylamide group forming a covalent interaction with the allosteric Cys23. Notably, this covalent binding mechanism allows EN523 to engage OTUB1 without inhibiting its deubiquitinating enzyme activity. Building on this discovery, the researchers conjugated EN523 to ΔF508-CFTR regulator Lumacaftor using C3 or C5 alkyl linkers, yielding heterobifunctional small molecules NJH-2-056 and NJH-2-057. Evaluation of these compounds in human bronchial epithelial cells expressing ΔF508-CFTR revealed that NJH-2-057 treatment produced substantial CFTR protein stabilization, evidenced by significant quantitative increases in mature CFTR levels.

Subsequently, research teams led by Professor Jin^4^ optimized the OTUB1 covalent ligand EN523 through structure‒activity relationship (SAR) studies, yielding MS5105. MS5105 employs a dimethylamino-2-enamide warhead, enabling faster OTUB1 modification (60% *vs* 20%) alongside improved water solubility (31.4 *vs* 0.8 mg/mL) and stability. To evaluate its potential in DUBTAC design, the team incorporated MS5105 as the OTUB1 recruiter and combined it with ΔF508-CFTR regulator Lumacaftor, synthesizing multiple DUBTACs *via* distinct linkage strategies. Among these, MS6178 exhibited superior performance, showing ∼10-fold enhanced stabilization of ΔF508-CFTR compared to NJH-2-057.

In April 2024, a research team led by Professor Wei^5^ reported the first DUBTAC leveraging deubiquitinase USP7. Departing from prior OTUB1-focused covalent ligands, this study employed a non-covalent USP7 inhibitor for DUBTAC design. Using USP7 ligand #1 (previously reported) as the deubiquitinase ligand, structural analysis of the USP7-ligand #1 co-crystal complex identified an amino group outside the binding pocket as the linker attachment site. Synthesizing DUBTACs through diverse linkers between ligand #1 and ΔF508-CFTR regulator Lumacaftor, the team evaluated their CFTR-stabilizing efficacy *in vitro*. Among these, MS6869 demonstrated CFTR stabilization comparable to NJH-2-057. These findings underscore DUBTAC’s transformative potential in biomedicine.

The following year, research led by Professor Wei^161^ on the co-crystal structure of USP28 and its non-covalent inhibitor KL9 (an analog of CT1073) revealed that the bridged piperidine moiety of KL9 binds to an allosteric site on the surface of USP28 and is solvent-exposed. Based on this observation, the team proposed that extending a linker from the bridged piperidine group could allow the resulting DUBTAC to retain binding to USP28 without inhibiting its deubiquitinase activity. Accordingly, they designed a series of USP28-based DUBTAC complexes by conjugating the non-covalent USP28 ligand CT1073 to the CFTR ligand lumacaftor *via* linkers of varying lengths. Among these, MS9279 exhibited efficacy comparable to the reported compound NJH-2-057. Further characterization demonstrated that MS9279 stabilized CFTR protein in a concentration- and time-dependent manner, with a sharp increase in CFTR levels observed after 18 h.

Professor Jin’s team^162^, through structural analysis of the non-covalent USP1 inhibitor ML323, revealed that the compound binds within the catalytic pocket of USP1 and possesses a solvent-exposed isopropyl group, making it suitable for linker attachment. However, DUBTACs derived from ML323 failed to stabilize ΔF508-CFTR, likely due to potent inhibition of USP1 (IC_50_ = 76 nmol/L). The team therefore switched to a weaker USP1 ligand, compound **55** (IC_50_ = 1.3 μmol/L), and conjugated it to lumacaftor using linkers of different lengths to construct a new series of molecules. Only compounds containing longer PEG linkers (PEG3–PEG5) significantly enhanced CFTR protein levels, with MS5310 being the most effective. Further studies indicated that MS5310 increased CFTR expression in a concentration- and time-dependent manner, with protein levels continuing to rise after 24 h. Its stabilizing effect surpassed those of previously reported CFTR-directed DUBTACs based on OTUB1 (MS6178) or USP7 (MS6869). Although proteomic analysis did not directly detect CFTR, significant upregulation of downstream pathway proteins, including HMGCS1, IDI1, and CYP51A1, was observed, indicating functional recovery of CFTR.

The strategy not only presents a groundbreaking therapeutic avenue for cystic fibrosis with clinical advancement implications but also establishes a framework for precision-targeted drug development. It enables overcoming traditional limitations to design potent, specific therapies.

Cyclic GMP–AMP synthase (cGAS) serves as a critical intracellular DNA sensor^163^. Detection of tumor-derived or tumor-associated viral DNA by cGAS activates its catalytic synthesis of cyclic GMP–AMP (cGAMP), which triggers STING pathway activation^164^. This signaling cascade induces type I interferon production and dendritic/macrophage maturation, enhancing antitumor immunity through improved pathogen recognition and clearance^165^. In chronic HBV infection models, cGAS expression is downregulated or functionally impaired, leading to deficient antiviral immune responses and viral persistence^166^. Persistent HBV replication drives chronic hepatic inflammation and fibrosis, culminating in cirrhosis and hepatocellular carcinoma in severe cases^167^^,^^168^. Several PROTACs with potent antiviral activity have been successfully developed, including those targeting human cyclophilin A (CypA). Studies have demonstrated that these CypA-directed PROTACs specifically reduce CypA levels in cell lines and primary human cells, exhibit superior anti-HIV-1 activity compared to the parent inhibitor in primary human CD4^+^ T cells, and suppress HCV replicons in hepatocellular carcinoma cell lines^169^. Given cGAS’s pivotal role in antitumor and antiviral defense—and the pathological consequences of its dysfunction—therapeutic strategies to stabilize and activate cGAS protein are urgently needed.

Building on these findings, research teams led by Professors Jin^4^ developed the first cGAS-targeted DUBTACs through a DUBTAC-based strategy. Researchers determined the co-crystal structure of cGAS in complex with G108 (an analog of G140) using X-ray crystallography and cryo-electron microscopy^170^. Based on these structural insights, the most potent cGAS inhibitor, G140, was selected as the targeting ligand. A series of linkers with varying lengths were systematically attached to the pyrazole ring of G140 *via* amide or retro-amide bonds to investigate the effect of linker chemistry on ternary complex formation and degradation efficiency. The optimized constructs were subsequently conjugated to the 5-methylthio benzoic acid/amine group of the OTUB1 ligand MS5105, culminating in the synthesis of four distinct classes of DUBTAC molecules. Screening identified two lead compounds, MS7829 and MS8588 (9-carbon linkers), that elevated cGAS protein levels 3- to 4-fold in a dose- and time-dependent manner while inducing 2- to 3-fold STING upregulation, effectively suppressing cancer cell proliferation and colony formation^4^. Given the cGAS/STING pathway’s critical role in antitumor immunity, these DUBTACs represent a novel immunotherapeutic avenue for cancer treatment.

The following year, the research team conjugated the cGAS antagonist G140 with the USP28 non-covalent ligand CT1073 to generate a series of USP28-based cGAS DUBTACs (designated as GDT compounds) and systematically evaluated their cGAS-stabilizing effects in HeLa cells. MS2099 and MS2100 were identified as the most potent compounds within the series, effectively elevating cGAS protein levels. In time-course experiments, MS2100 significantly increased both cGAS and its downstream effector STING within 3 h of treatment. The team further assessed the anti-proliferative activity of MS2099 and MS2100 using live-cell imaging and colony formation assays. The results demonstrated that both compounds markedly inhibited the growth of HeLa cells, even reducing cell numbers below the initial seeding density^161^.

AMPK is a central therapeutic target in type 2 diabetes research^171^. As a master intracellular energy sensor, AMPK dynamically regulates cellular metabolism by modulating glucose uptake, lipid catabolism, and insulin signaling pathways^111^^,^^172^. Upon activation, AMPK induces glucose transporter type 4 (GLUT4) translocation from intracellular storage to the cell membrane, enhancing glucose uptake in muscle and adipose tissues^173^^,^^174^. Mechanistically, AMPK phosphorylates critical nodes in insulin signaling, such as IRS-1, thereby amplifying insulin sensitivity and glucose homeostasis^175^^,^^176^. Target protein downregulation should be primarily driven by UPS-mediated degradation, rather than reduced transcription or translation, as this premise constitutes a fundamental basis for the rational design of DUBTACs. In pathological contexts where target protein stability is dynamically regulated by the UPS, rebalancing protein levels through deubiquitination holds clear therapeutic relevance. The degradation of AMPK*α* under high glucose exemplifies this concept, high glucose promotes ROS generation, which activates AKT. AKT then phosphorylates AMPK*α* at serines 485 or 491, triggering recruitment of the skeletal muscle-enriched E3 ubiquitin ligase MG53. This leads to ubiquitination at lysine 470 of AMPK*α* and consequent reduction in its protein levels^177^^,^^178^. While small-molecule AMPK agonists demonstrate symptomatic improvements, their clinical utility is constrained by suboptimal specificity and off-target effects.

The DUBTAC strategy demonstrates significant potential for developing optimized therapeutics. Professor Wei^179^ engineered USP7-targeted DUBTACs by conjugating the non-covalent USP7 inhibitor USP7 ligand #1 with the AMPK agonist 991. Cellular assays revealed that these DUBTACs reduced AMPK*β*1 ubiquitination while enhancing phosphorylation of its downstream substrate acetyl-CoA carboxylase (ACC). Oil Red O staining quantification further demonstrated that DUBTAC treatment decreased intracellular lipid accumulation, confirming functional AMPK*β*1 stabilization. These findings establish that USP7-targeted DUBTACs effectively stabilize AMPK*β*1/*β*2, activate AMPK signaling, and suppress tumor cell proliferation^5^.

In recent years, PPAR*γ* has emerged as a promising therapeutic target in oncology. Studies show that its activation suppresses proliferation, induces apoptosis, and reduces migratory and invasive capacities of cancer cells^180^. PPAR*γ* activation also remodels the tumor microenvironment (TME) and enhances antitumor immunity, thereby impairing tumor growth and metastasis^181^. In many solid tumors, including lung, breast, and colorectal cancers, PPAR*γ* protein expression is frequently downregulated^182^. Notably, in non-small cell lung cancer (NSCLC), low PPAR*γ* levels correlate with advanced stage, increased invasiveness, and poor prognosis. Conventional PPAR*γ* agonists are limited clinically by systemic metabolic side effects, highlighting the need for alternative strategies such as DUBTACs^183^^,^^184^.

In a 2025 study, Professor Wei’s^161^ team elucidated the co-crystal structure of PPAR*γ* bound to the agonist lobeglitazone (LGZ), which revealed a solvent-exposed methoxy group on LGZ’s phenyl ring suitable for linker conjugation. Leveraging this insight, they derivatized LGZ at this position and connected it to CT1073, a non-covalent USP28 ligand, *via* linkers of varying lengths to generate a series of PPAR*γ*-directed DUBTACs. Screening in HeLa cells identified MS1727–MS1730 as potent stabilizers of PPAR*γ*. These compounds consistently enhanced PPAR*γ* levels in both HeLa and triple-negative breast cancer MDA-MB-231 cells. Competitive rescue experiments confirmed target engagement: pre-treatment with free CT1073 or LGZ abolished MS1728-mediated stabilization. These results demonstrate that MS1727–MS1730 promote PPAR*γ* stabilization through specific recruitment to USP28, leading to deubiquitination, reduced proteasomal degradation, and enhanced functional protein expression.

DUBTAC technology represents a transformative therapeutic platform, demonstrating efficacy in rebalancing proteostasis across cystic fibrosis, cancer, and metabolic disorders. By enabling spatiotemporal regulation of ubiquitination dynamics, this approach facilitates functional restoration of pathologically dysregulated proteins—a paradigm-shifting strategy for targeting “undruggable” disease mechanisms. Many proteins with critical biological functions are expressed at low abundance under pathological conditions. For instance, cGAS is downregulated during chronic HBV infection, and AMPK expression is reduced in metabolic tissues of individuals with type 2 diabetes, thereby limiting engagement by small-molecule ligands. Consequently, the effective targeting of low-abundance proteins requires that DUBTAC ligands exhibit high binding affinity to enable efficient ternary complex formation and functional activity. Inadequate ligand affinity may result in failure to recognize and stabilize the target, significantly compromising stabilization efficiency. Thus, during the development of DUBTACs directed against low-abundance targets, the evaluation of binding efficiency at physiologically relevant low concentrations should be a central criterion in lead optimization to ensure robust efficacy under target-limited pathological conditions.

## 6 Possibility of nucleic acids, peptides or antibodies as warheads

**Figure 7:**
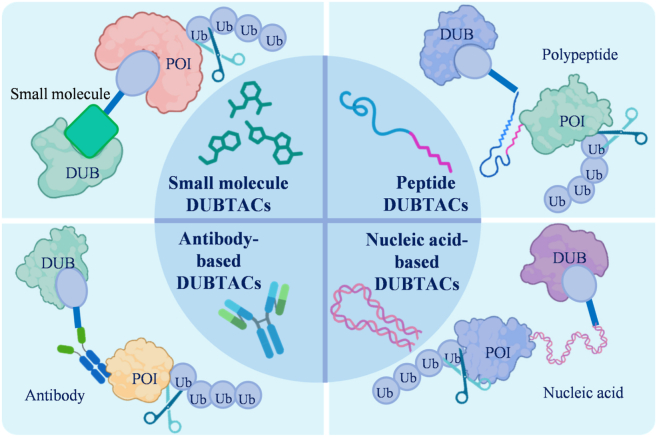
NSM–DUBTACs. Given the absence of suitable small molecule agonists for many target proteins, noncanonical DUBTAC platforms (polypeptide-/antibody-/nucleic acid-based) offer viable design alternatives.

DUBTAC technology development remains nascent, with current warheads primarily limited to small molecules or nucleic acids. Leveraging PROTAC-proven strategies through their technical parallels, we propose designing analogous antibody- or nucleic acid-based DUBTACs (Fig. 7). This review outlines approaches to enhance targeted protein stabilization toolkits, citing developed bifunctional DUBTACs (*e.g.*, targeting ΔF508-CFTR and AMPK) that significantly upregulate their respective proteins. In the realm of nucleic acid DUBTACs, teams led by Professor Wei^91^ pioneered TF-DUBTAC platforms using transcription factor (TF)-specific DNA motifs to stabilize tumor suppressors. Their FOXO-, p53-, and IRF-DUBTACs maintain FOXO3A, p53, and IRF3 stabilization *via* OTUB1-dependent mechanisms, establishing a generalizable strategy for TF-targeted therapies.

While peptide- or antibody-based DUBTACs remain unreported, established PROTAC strategies offer actionable insights for their design. Sakamoto et al.^185^ developed a peptide PROTAC targeting the androgen receptor (AR), inducing AR degradation and suppressing AR-dependent proliferation. Edward W. Tate’s team^186^ engineered an antibody-based PROTAC (Ab-PROTAC) by conjugating bromodomain protein 4 (BRD4)-targeting PROTACs to trastuzumab (anti-HER2), achieving BRD4 degradation in human epidermal growth factor receptor 2 (HER2) + breast cancer cells. These approaches exemplify the potential of expanding molecular formats to enable targeted delivery, a concept that may also inform DUBTAC development. However, extending the Ab-PROTAC strategy to Ab-DUBTAC presents considerable challenges. Unlike PROTACs, which promote degradation, DUBTACs are designed to restore target protein function. This requires that the deubiquitinated protein not only avoids degradation but also achieves proper folding, traffics correctly to its functional compartment, such as the nucleus, mitochondria, or plasma membrane, and regains biological activity. As a result, the feasibility of Ab-DUBTACs is likely constrained by the subcellular localization behavior of the target protein, limiting broad applicability. Their development will therefore critically depend on advances in delivery technologies and improved understanding of protein refolding and localization mechanisms. These PROTAC-inspired DUBTAC modalities hold potential to overcome traditional drug limitations, offering novel therapeutic avenues for cancer, autoimmune disorders, and neurodegeneration.

## 7 Summary

As an emerging technology, DUBTAC remains considerably less developed than PROTAC. This article reviews DUBTAC from multiple perspectives, examining its successful application in models of cystic fibrosis, cancer, and metabolic diseases, and highlighting its therapeutic efficacy and broad potential. It is noteworthy that most current studies still utilize DUB inhibitors as recruiting ligands for DUBTAC assembly. Although these constructs enhance target protein stability, the mechanistic underpinnings remain poorly understood, and potential functional interference cannot be ruled out. Future design strategies should prioritize the development of DUB-agonist ligands that enhance deubiquitination activity, enabling more precise and physiologically compatible protein stabilization. This shift represents a critical direction for advancing the rational design of DUBTAC technology.

So far, reported applications of DUBTACs have been limited to only a few DUBs, such as USP7, OTUB1, and USP1, with no systematic exploration across major DUB families including USP, OTU, and JAMM. More importantly, the field still lacks clear functional and structural criteria to identify which DUBs are suitable for recruitment in DUBTACs and which are mechanistically or biologically constrained. Nevertheless, advances in structural biology offer a foundation for rational DUBTAC design. High-resolution co-crystal structures of targets like ΔF508-CFTR and cGAS have informed ligand selection and linker attachment strategies. Notably, structural insights have been indispensable in elucidating molecular recognition—as seen in PROTACs, where VHL and CRBN complex structures revealed key interaction residues and mechanisms of ubiquitin ligase recruitment and degradation. Similarly, DUBTAC efficacy depends on precise spatial compatibility and cooperative binding among the target, DUBTAC, and DUBs. Yet, no ternary complex structure (target–DUBTAC–DUBs) has been resolved, greatly limiting understanding of conformational dynamics, interface recognition, and stabilization mechanisms. Here, AI holds great potential. Beyond adopting PROTAC-derived machine learning models for ligand prediction, linker design, and ADMET profiling, emerging tools such as AlphaFold-Multimer show high accuracy in predicting protein–protein and multicomponent complex structures^187^, ^188^, ^189^. These approaches may help simulate DUBTAC ternary complexes, propose binding modes, and identify spatial constraints. When integrated with sparse experimental data, AI can suggest optimal linker geometries and assess complex stability, thereby compensating for the lack of empirical structures and facilitating a transition from empirical screening to data-driven, structure-guided rational design of DUBTACs.

As an emerging therapeutic modality, DUBTACs hold considerable promise for stabilizing dysfunctional target proteins. However, their clinical translation faces several challenges. First, heterogeneity in target protein turnover rates may significantly affect DUBTAC efficacy, and the stabilization of rapidly cycling proteins requires further systematic validation. Second, the tissue-restricted expression patterns of many deubiquitinating enzymes raise concerns regarding potential organ-specific toxicity upon off-target activation, underscoring the need for careful DUBs selection and profiling. Moreover, DUBTAC development demands integrated optimization of multiple parameters, including the catalytic efficiency of the recruited DUBs, ligand selectivity, and drug-like properties such as membrane permeability and metabolic stability—all of which collectively determine compound feasibility. Future efforts should focus on stabilization efficiency, phenotypic predictability, target specificity, and off-target risks through systematic experimentation and multidimensional optimization to facilitate the transition of DUBTACs from concept to clinic.

## Author contributions

Original draft preparation: Danfeng Wang, Wenjian Min and Binjian Jiang; review and editing: Haopeng Sun, Chengliang Sun and Peng Yang.

## Conflicts of interest

The authors declare no conflicts of interest.
